# Childhood Adiposity and Bisphenol Exposure Are Linked to Gut Dysbiosis, Altered Short-Chain Fatty Acid and Complex Carbohydrate-Degrading Profiles

**DOI:** 10.3390/nu18213560

**Published:** 2026-10-28

**Authors:** Pilar Ortiz, Esther Palacios, Ana Moreno-Blanco, Alicia Ruiz-Rodríguez, Margarita Aguilera

**Affiliations:** 1 Department of Microbiology, Faculty of Pharmacy, University of Granada, 18071 Granada, Spain; aliruizrodriguez@ugr.es (A.R.-R.); maguiler@ugr.es (M.A.); 2 Human Microbiota Laboratory, Institute of Nutrition and Food Technology “Jose Mataix Verdú”, Biomedical Research Center, Health Sciences Technological Park, University of Granada, 18016 Granada, Spain; 3 Microbiology Department, University Hospital Ramón y Cajal, Ramón y Cajal Health Research Institute (IRYCIS), 28034 Madrid, Spain; esthepalgo@gmail.com (E.P.); anamorenoblanco1993@gmail.com (A.M.-B.); 4 Biomedical Research Networking Center for Infectious Diseases (CIBERINFEC), Carlos III Health Institute, 28034 Madrid, Spain; 5 Institute of Biosanitary Research of Granada IBS, 18012 Granada, Spain

**Keywords:** short-chain fatty acids (SCFA), carbohydrate metabolism, bisphenols, adiposity, obesity, microbiota

## Abstract

Background: Dietary exposure to endocrine-disrupting chemicals is increasingly associated with pediatric adiposity and gut dysbiosis. Although short-chain fatty acids (SCFAs) are traditionally known for being involved in metabolic homeostasis, their contribution to energy harvesting remains controversial and under continuous debate. Methods: We conducted an integrative study of 100 children (aged 3–12 years) stratified by BMI and total body fat percentage, with bisphenol exposure assessed via nail matrix biomonitoring. Gut microbiota was profiled via amplicon gene sequencing, fecal SCFAs were quantified by GC-MS, and the genomic capacity for complex carbohydrate degradation was predicted using functional profiling tools. Results: Children with high body fat percentage exhibited higher fecal concentrations of acetate, butyrate, and propionate. High bisphenol levels were also positively associated with both increased adiposity and elevated fecal SCFA profiles. Integrative analyses identified a distinct microbiota-metabolic signature: obesity and high bisphenol exposure were linked to an expansion of specific taxa such as *Blautia*, *Collinsella*, and *Catenibacterium*, contrasting with a reduction in potential protective markers including Oscillospiraceae, *Bacteroides plebeius*, and Christensenellaceae R-7. Notably, key functional and gene-enzymatic profiling revealed an enriched carbohydrate-degrading potential within the adiposity and BPA-associated microbiota, consistent with enhanced fiber degradation, increased SCFA production, and greater predicted energy-extracting potential. Conclusions: The co-occurrence of host metabolic and environmental factors together with the altered microbiome features, points towards a shift in microbial energy harvesting. The findings outline a correlative ‘chemical obesogen–microbiome–adiposity’ interplay, linking environmental xenobiotic exposure to specific microbial alterations and metabolic changes associated with pediatric fat accumulation.

## 1. Introduction

The prevalence of childhood obesity has reached epidemic levels, witnessing an almost ten-fold increase over the past four decades. According to the World Health Organization, nearly 390 million children and adolescents aged 5–19 are currently affected by overweight or obesity globally [1]. This trend is particularly concerning because pediatric obesity is a major precursor to chronic and non-communicable diseases such as type 2 diabetes and cardiovascular disorders in early adulthood [2]. While this metabolic alteration, characterized by an excessive fat deposition [1,3], is driven by a complex interplay of genetic, dietary, and environmental factors [4], recent advances in high-throughput sequencing data have highlighted the gut microbiota as a critical mediator within this framework, acting as a central regulator of host energy metabolism and adipose tissue function [5].

A major mechanism through which the gut microbiota influences host metabolism is the production of microbial-derived metabolites or co-metabolites, particularly short-chain fatty acids (SCFAs), such as acetate, propionate, and butyrate [6]. These metabolites are primarily derived from the fermentation of non-digestible dietary fibers by specific colonic bacteria. Upon reaching the systemic circulation, SCFAs act as crucial signaling mediators with well-documented health-promoting impact, including anti-inflammatory effects, enhancement of insulin sensitivity, stimulation of hepatic fat oxidation, and promotion of satiety [7]. Despite this health-promotion, the physiological routes, mechanisms and biological impact of SCFAs is complex and controversial. In state of chronic and specific dysbiosis, altered SCFA production and absorption profiles have been linked to enhanced energy harvest from diet and to metabolic disturbances favoring adipose tissue expansion [8]. It has been proposed that a high abundance of carbohydrate-fermenting bacteria may contribute to the pathogenesis of obesity by increasing SCFA biosynthesis. This process provides an additional energy source for the host that is eventually stored as lipids or glucose, thereby facilitating the transition toward an obese phenotype [9]. 

Beyond dietary and endogenous factors, environmental exposure to endocrine-disrupting chemicals (EDCs) has emerged as a significant driver of the global obesity crisis and as a long-term modifier of the gut microbiome [10,11]. Among these, bisphenol A (BPA) is a widespread environmental obesogenic contaminant, largely due to its extensive application in the manufacture of plastics and epoxy resins [12]. Growing evidence suggests that bisphenols, particularly during early life, may reshape the gut microbial landscape, potentially diminishing the abundance of beneficial commensals while enriching taxa associated with more efficient energy harvesting from the diet [13,14]. Given their capacity for maternal-infant transfer during critical developmental windows [15], these contaminants severely threaten early microbiome maturation and host energy regulation. To accurately understand their impact on pediatric metabolic health, chemical nail deposits have emerged as reliable and stable matrices for biomonitoring chronic early-life exposure [16].

In this complex context, the present study utilizes an integrative approach combining 16S rRNA microbiome profiling, targeted SCFA metabolomics, and predictive functional genomics of carbohydrate-degrading capacity in children with normal weight, overweight and obesity. By integrating these data with quantified bisphenol levels from keratinized matrices, we aim to elucidate microbiota-metabolism mediated signatures linking early-life environmental chemical exposure to obesity risk.

## 2. Materials and Methods

### 2.1. Study Cohort

Children (*n* = 100) between the ages of 3 and 12 were recruited from an observational study approved by the Institutional Ethic Committee from the University of Granada (CEIM/CEI 23/12/2019-Acta12/19). All studies were conducted in accordance with the ethical principles of the Declaration of Helsinki. Prior to study enrollment, formal written informed consent was obtained from the parents or legal guardians of all participating children. To ensure the integrity of the microbial data, only children without intestinal disorders and who had not received antibiotic treatment within the three months preceding the study were included. Anthropometric measurements were collected according to the study guideline [17]. Participants were classified into weight status categories based on age- and sex-specific Body Mass Index (BMI) cut-off points, following the International Obesity Task Force (IOTF) criteria as defined by Cole et al. [18]. Body composition was assessed using a bioelectrical impedance analyzer (Tanita MC-780 S MA, TANITA Corporation, Tokyo, Japan), measuring fat mass (% and kg), muscle mass (% and kg), and total body water (% and kg). Bisphenol biomonitoring data from individual finger- and toenail samples were available for a subset of 42 children from the cohort. Analyte determination was performed following the validated methodology described by Martín-Pozo et al. [19]. Briefly, nail samples were first washed to remove external contamination, then subjected to alkaline digestion, and subsequently analysed by ultra-high-performance liquid chromatography coupled to tandem mass spectrometry (UHPLC–MS/MS) using an Acquity UPLC™ I-Class system equipped with a Waters UPLC^®^ BEH C18 column (2.1 mm × 50 mm, 1.7 μm particle size) coupled to a Xevo TQ-XS triple quadrupole mass spectrometer (Waters, Manchester, UK). Bisphenol A (BPA) and bisphenol F (BPF) were the analytes consistently detected and quantified in the nail matrix. The limits of detection (LOD) and quantification (LOQ) were 1 ng g^−1^ and 4 ng g^−1^, respectively, for both BPA and BPF. Total bisphenol exposure (BIS) was calculated as the sum of all quantified analytes; values below the limit of detection (LOD) were imputed as LOD/√2 per EFSA guidelines [20].

### 2.2. Fecal Sample Collection and DNA Extraction

Fecal samples from each participant were freshly and anaerobically collected and maintained frozen at −80 °C until further laboratory processing. DNA extraction was performed using the PowerSoil DNA Isolation Kit (Qiagen^®^, Hilden, Germany) following the manufacturer’s instructions. The isolated DNA was sent to Novogene Europe (Cambridge, UK & Munich, Germany Omics Centers) for library preparation and subsequent high-throughput sequencing. To characterize the gut microbiota, the V3-V4 amplicon hypervariable region of the prokaryotic 16S rRNA gene was targeted for microbial sequencing and profiling. Amplification was performed using the universal primer pair 341F (5′-CCTAYGGGRBGCASCAG-3′) and 806R (5′-GGACTACNNGGGTATCTAAT-3′), incorporating a dual-barcoding strategy for sample multiplexing. Sequencing libraries were constructed using the NEBNext^®^ Ultra™ II FS DNA PCR-free Library Prep Kit (New England Biolabs, Ipswich, MA, USA). To ensure the integrity of the results, library quality was rigorously validated through Qubit fluorometric quantification, real-time PCR, and bioanalyzer assessment, confirming optimal fragment size and concentration. Finally, the pooled libraries were sequenced on the Illumina NovaSeq 6000 platform. The DADA2 pipeline [21] was utilized for denoising and sequencing error correction, resulting in the identification of Amplicon Sequence Variants (ASVs), which were then taxonomically classified using the QIIME2 [22]. Sequences identified as non-bacterial or falling below a 0.1% relative abundance threshold in fewer than two samples were removed from downstream analysis.

### 2.3. Short-Chain Fatty Acids Quantification

Fecal levels of SCFAs were determined in cell-free fecal supernatants prepared as previously described [23] by Gas Chromatography coupled to Mass Spectrometry (GC-MS) at the Ramón y Cajal Health Research Institute (IRYCIS). GC-MS analysis was performed using a TRACE 1600/1610 gas chromatograph/ISQ7610 mass selective detector (Thermo Fisher Scientific, Dreieich, Germany) equipped with a TG-WaxMS A GC Column (15 m × 0.32 mm × 0.25 μm, Thermo Fisher Scientific, Dreieich, Germany). SCFAs and their target (TI) and confirmative ion (CI) *m*/*z* are listed in Supplementary Table S1. Compounds were identified against the NIST database and confirmed by the retention times of pure standards. The specific analytes were detected in selected ion monitoring (SIM) mode and quantified using deuterated butyric acid as internal standard and external calibration curves. The instrument was operated, and the data was acquired and analyzed using Chromeleon 7 software.

### 2.4. Data Processing and Statistical Analysis

The study integrated multiple datasets, including anthropometric measurements (BMI, body fat percentage), fecal SCFAs concentrations, gut microbiota taxa and gene profiles, and nail bisphenol exposure markers (BPA, BPF, and total bisphenols). All analyses were conducted in the R statistical computing environment.

Continuous variables were assessed for normality. Parametric data are presented as mean and standard deviation (SD), while non-parametric variables are expressed as median and interquartile range (IQR). Categorical variables, such as sex distribution, were summarized by frequencies and analyzed using Pearson’s Chi-squared test. Intergroup differences in clinical and metabolic parameters were evaluated using the Kruskal-Wallis test, followed by Dunn’s post-hoc test with Benjamini-Hochberg (BH) correction for multiple comparisons. To investigate the influence of adiposity, the cohort was stratified based on the global median of body fat percentage. This also enabled the identification of the already known ‘Normal Weight Obesity’ (NWO) phenotype, individuals within the NW range by BMI but exceeding the body fat median [24]. The relationship between adiposity and SCFA levels was further assessed through Spearman’s rank correlation. Importantly, the potential influence of biological confounders, specifically age and sex, was independently evaluated to ensure that these factors did not significantly bias the observed metabolic profiles. Given the significant sex differences observed across BMI groups, sex was included as a covariate in all relevant statistical models.

Microbial community characterization was performed through several approaches: (i) alpha diversity was estimated using the Observed taxa, Shannon, and Inverse Simpson indices, (ii) beta diversity was visualized via Principal Coordinates Analysis (PCoA) based on Bray-Curtis distances, with community dissimilarity across BMI and bisphenol exposure groups tested via Permutational Multivariate Analysis of Variance (PERMANOVA), and (iii) differential abundance analysis at the Amplicon Sequence Variants (ASVs), genus, and phylum levels was conducted using the MaAsLin2 R package, adjusting for potential confounders such as sex where appropriate.

To explore the interplay between the taxa and metabolite microbiota data, environmental exposure and host adiposity, the following integrative methods were employed: (i) Spearman’s rank correlations were used to associate fecal SCFA levels with differentially abundant ASVs, (ii) Mantel tests were performed to assess community-level relationships between microbial signatures, bisphenol concentrations, and obesity indices, (iii) distance-based Redundancy Analysis (dbRDA) was implemented to identify the environmental and host factors (bisphenols, adiposity, and SCFAs) that significantly drove the variance in microbial community structure, and (iv) the carbohydrate-degrading potential of differential key ASVs was evaluated by querying their 16S rRNA sequences via BLASTn to identify the closest related strain with an available complete genome assembly. Subsequently, these retrieved reference assemblies were analyzed using the dbCAN2 meta-server [25] to annotate relevant Carbohydrate-Active enZyme (CAZyme) families according to the CAZy database. This comparative genomic approach allowed us to estimate the functional capacity of these taxa to degrade six major dietary substrate families (starch, cellulose, hemicellulose, arabinan, pectin, and inulin/fructooligosaccharides (FOS).

## 3. Results

### 3.1. Pediatric Cohort Characteristic Description

General characteristics of the pediatric cohort are summarized in Table 1. The 100 children were classified according to IOTF criteria into three groups: normal weight (NW; *n* = 47), overweight (OW; *n* = 16), and obese (O; *n* = 37). While no significant variations in age were observed among the study groups, sex distribution differed significantly, characterized by a higher female prevalence in the NW and OW categories relative to the O group. 

### 3.2. Characterization of the Gut Microbiota

**Figure 1:**
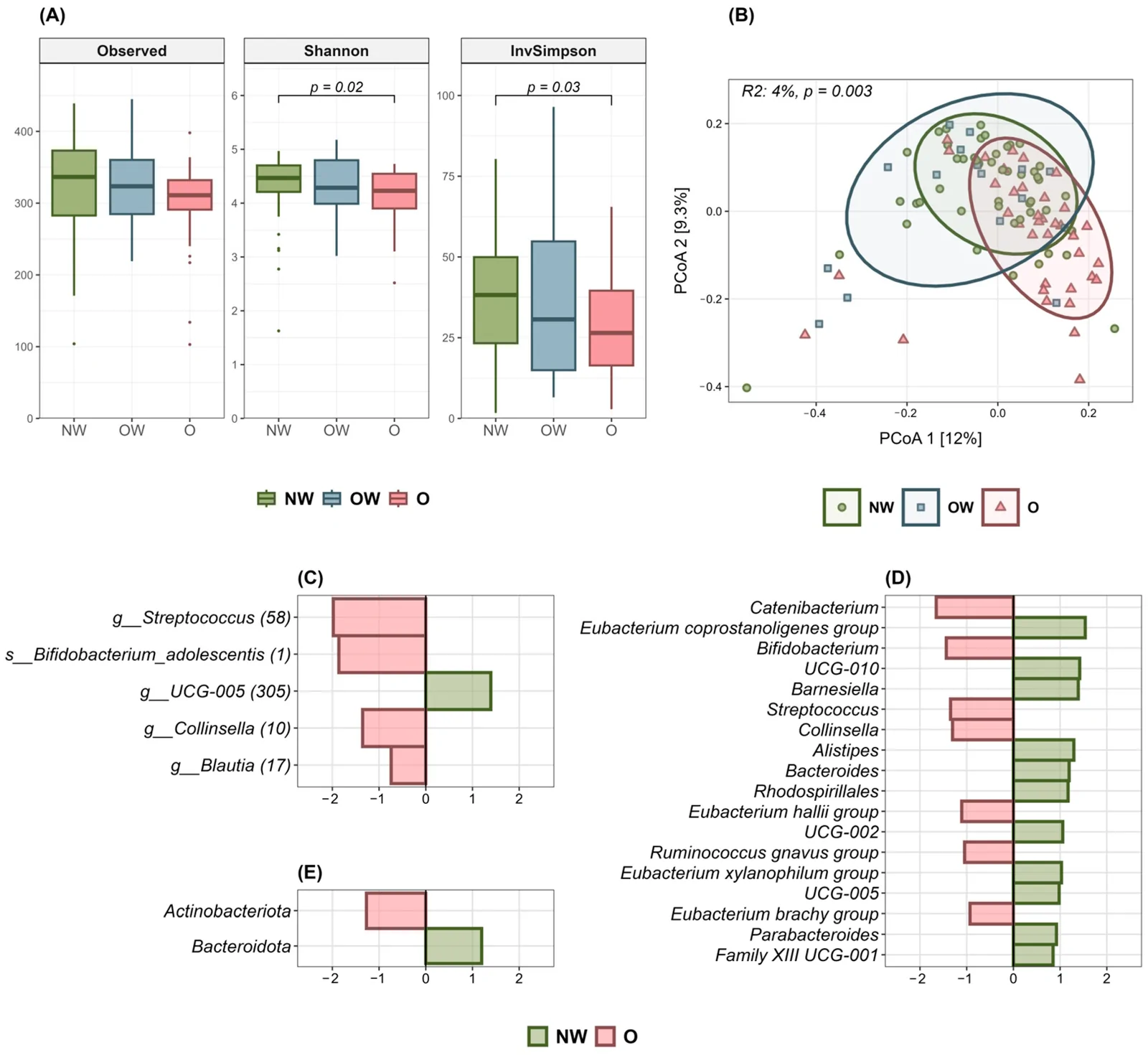
Global overview of gut microbiota composition in relation to BMI status. (**A**) Alpha diversity indices including Observed, Shannon, and Inverse Simpson. (**B**) Principal Coordinate Analysis (PCoA) based on Bray-Curtis dissimilarity showing the microbial community composition (beta diversity) of the study cohort. Differentially abundant ASVs (**C**), genera (**D**), and phylum (**E**) in association with BMI. Bar plots depict MaAsLin2 analysis results adjusted for sex. Significant results are shown (Benjamini-Hochberg corrected q < 0.25).

Intestinal microbial profiling revealed significantly reduced alpha diversity in the obesity (O) group compared to normal-weight (NW) participants (Shannon, *p* = 0.02; InvSimpson, *p* = 0.03; Figure 1A), while the overweight (OW) group showed no significant deviations. Beta diversity analysis confirmed significant compositional changes across BMI categories (PERMANOVA, *p* = 0.003; Figure 1B). Pairwise comparisons demonstrated this divergence was driven exclusively by the O group, which structurally differed from both NW and OW children (*p* = 0.001), whereas NW and OW did not significantly differ.

Consistent with diversity metrics, significant taxonomic shifts occurred exclusively between NW and O children. Obesity was marked by the enrichment of *Bifidobacterium adolescentis*, *Blautia*, *Streptococcus*, and *Collinsella* ASVs (Figure 1C), mirroring generic-level expansions of *Bifidobacterium*, *Collinsella*, and *Streptococcus* (Figure 1D). Conversely, NW microbiota was characterized by higher abundances of *Alistipes*, *Bacteroides*, *Barnesiella*, *Parabacteroides*, and Oscillospiraceae members (UCG-005, UCG-002). Overall, childhood obesity presented a fundamental structural reorganization driven by the expansion of the Actinomycetota phylum and the concurrent depletion of Bacteroidota (Figure 1E).

### 3.3. Quantitative Results of Fecal SCFAs

**Figure 2:**
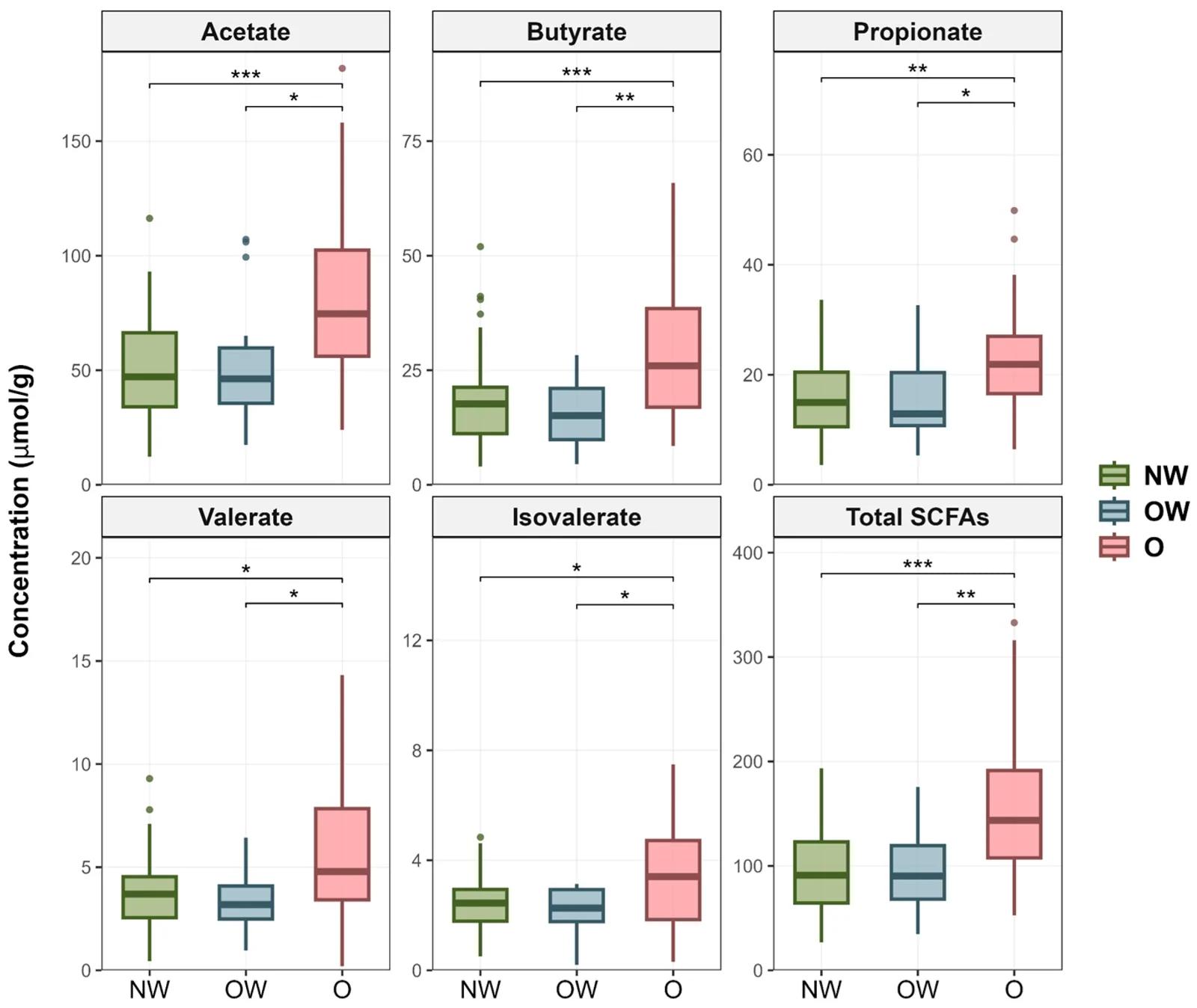
Fecal levels of SCFAs across BMI categories. Boxplots display concentrations (μmol/g) for metabolites reaching statistical significance after FDR correction (*p* < 0.05). The boxes represent the median and interquartile ranges for normal weight (NW), overweight (OW), and obese (O) groups. Pairwise comparisons were performed using Dunn’s post-hoc test; * indicates 0.01 < *p* value ≤ 0.05, ** indicates 0.001 < *p* value ≤ 0.01, and *** indicates *p* value ≤ 0.001.

The analysis of fecal SCFAs revealed significant differences across BMI groups (Figure 2), driven primarily by a higher total SCFA concentration in the O group compared to both NW (*p* < 0.001) and OW children (*p* = 0.004). Detailed median concentrations and standard deviations for each targeted metabolite across the study groups are summarized in Supplementary Table S2. Specifically, children in the O group exhibited significantly higher concentrations of the most abundant SCFAs, including acetate (*p* = 0.001), butyrate (*p* = 0.001), propionate (*p* = 0.005), valerate (*p* = 0.014), and isovalerate (*p* = 0.022). Post-hoc comparisons confirmed that this metabolic profile was specifically characteristic of the obese phenotype, as no significant differences were detected between the NW and OW groups for any of the analyzed SCFAs. Importantly, no significant differences were found between age and SCFA levels, and sex did not significantly influence the concentrations of these metabolites.

Furthermore, to link this metabolic profile with specific microbial signatures, Spearman’s rank correlations were performed between fecal SCFA concentrations and the five ASVs previously identified as differentially abundant between NW and O groups by MaAsLin2 analysis (Supplementary Figure S1). The NW-enriched taxa UCG-005 (305) displayed consistent inverse relationships across all major SCFAs. Conversely, the obesity-enriched taxa drove positive associations: acetate was positively correlated with all four obesogenic ASVs (*Blautia* 17, *Streptococcus* 58, *Bifidobacterium adolescentis* 1, and *Collinsella* 10), while propionate and butyrate exhibited positive correlations with *Streptococcus* (58) and *Blautia* (17), respectively.

### 3.4. Analysis of Fecal SCFAs and Bisphenol Levels Across Adiposity Phenotypes

**Figure 3:**
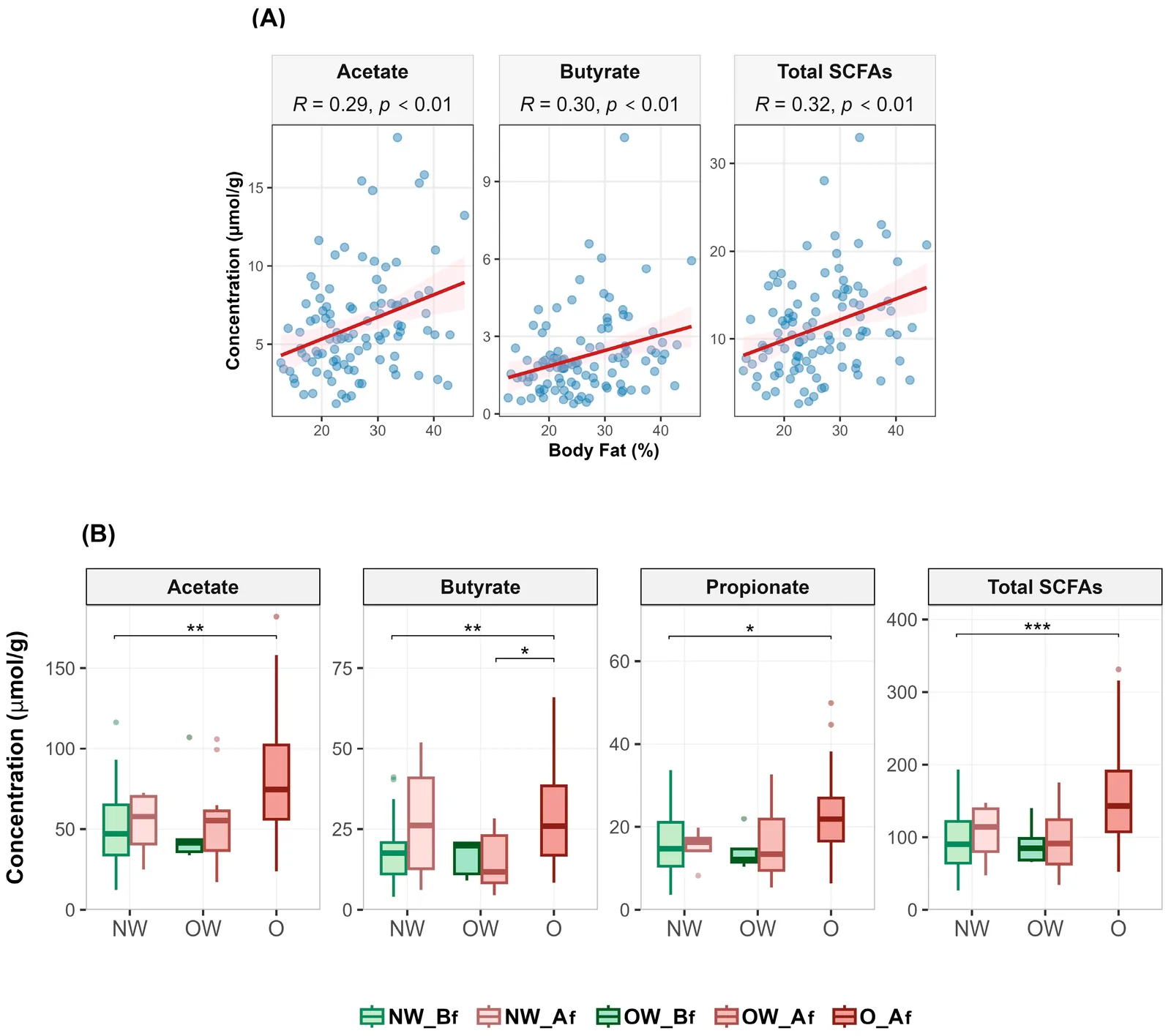
Association between adiposity markers and SCFA profiles. (**A**) Spearman correlation analysis between body fat percentage and fecal concentrations (μmol/g) of acetate, butyrate and total SCFAs. The red line represents the linear regression trend, and the shaded area indicates the 95% confidence interval. Spearman’s rank correlation coefficient (R) and *p*-values are displayed for each metabolite. (**B**) SCFA profiles stratified by BMI (Normal-Weight, NW; Overweight, OW; Obesity, O) and further subdivided by the global median body fat percentage (Below fat, Bf; Above fat, Af). Statistical significance was assessed via Dunn’s post-hoc test with Benjamini-Hochberg FDR correction (* *p* < 0.05, ** *p* < 0.01, *** *p* < 0.001).

Beyond categorical BMI groups, continuous body fat percentage exhibited significant positive correlations with total SCFAs, acetate, and butyrate (*p* < 0.01 for all three; Figure 3A). Conversely, no significant correlations were detected for propionate, valerate, or other minor SCFAs.

To further explore the relationship between adiposity and fecal SCFA profiles, the study population was stratified into two categories—above or below the global median of body fat percentage. All individuals classified with obesity (O) consistently presented body fat levels above the global fat median. In contrast, the normal-weight (NW) and overweight (OW) groups were subdivided into four subgroups: normal-weight below fat median (NW_Bf), normal-weight above fat median (NW_Af), overweight below fat median (OW_Bf), and overweight above fat median (OW_Af). Individuals in the NW_Af group exhibited median acetate and butyrate concentrations that were markedly higher than those of their below-fat counterparts (NW_Bf, Figure 3B). Remarkably, the median butyrate concentration in the NW_Af group was essentially identical to that observed in the obesity group. Post-hoc analysis confirmed that total SCFA concentrations, alongside acetate, butyrate, and propionate, were significantly elevated in the O group compared to NW_Bf (*p* < 0.05). Furthermore, the O group showed significantly higher butyrate levels even when compared specifically to overweight children with high adiposity (OW_Af, *p* < 0.01).

To investigate whether environmental exposures paralleled this adiposity-driven metabolic profile, nail bisphenol levels were determined in a subset of children (*n* = 42), for whom nail matrix biomonitoring data were available. The microbiota composition of this subset mirrored that of the initial cohort (r = 0.895, R^2^ = 0.801, *p* < 0.001; Supplementary Figure S2), supporting its representativeness for bisphenol-related analyses. Detailed characteristics, bisphenol concentrations, and fecal SCFA profiles for the subset are summarized in Supplementary Tables S3 and S4, alongside a comprehensive overview of their gut microbiota diversity and composition in Supplementary Figure S3. Initially, evaluating bisphenol levels strictly across categorical BMI revealed a trend of cumulative exposure scaling with weight (Supplementary Figure S4). Although not reaching statistical significance due to the restricted sample size per group and the high inter-individual dispersion inherent to human biomonitoring, children with obesity displayed substantially higher median BPA, BPF, and total bisphenol (BIS) concentrations compared to the NW and OW groups.

**Figure 4:**
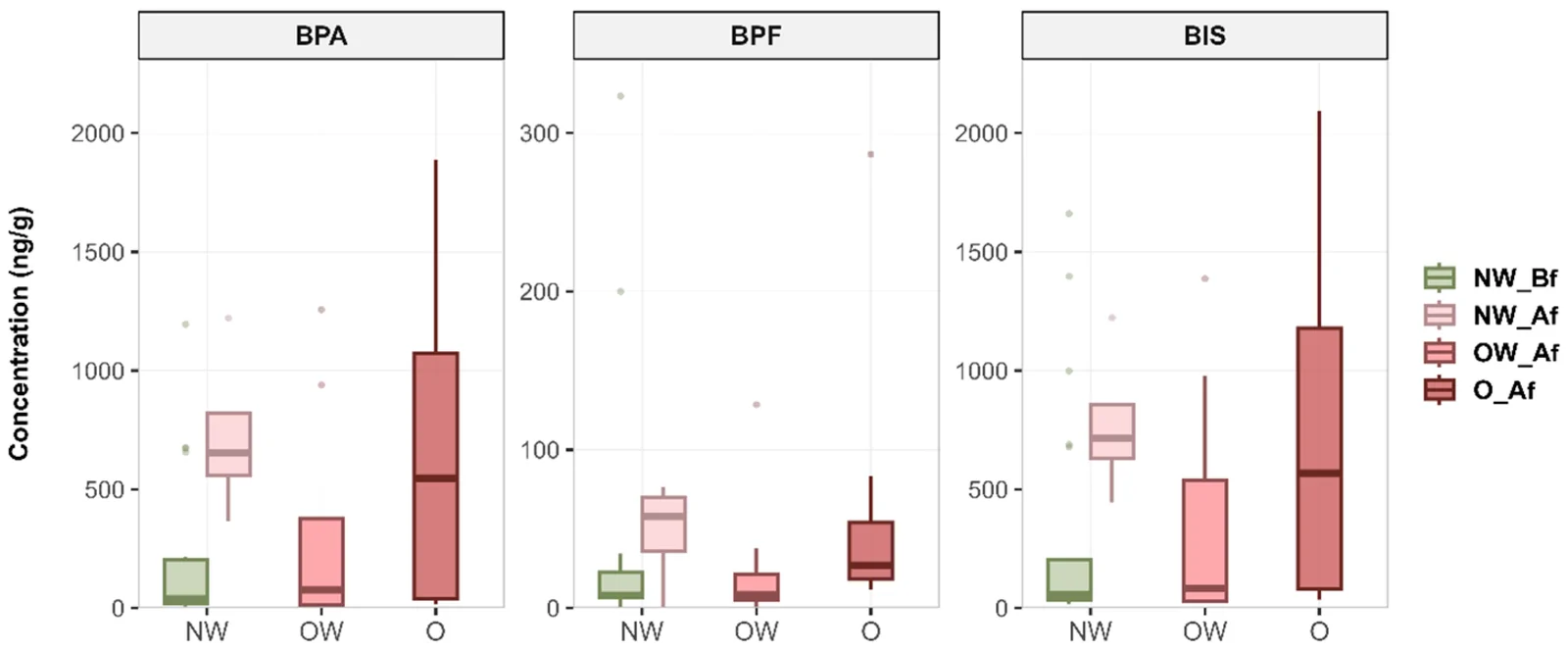
Bisphenol levels across BMI categories and body fat distribution. NW: normal weight; OW: overweight; O: obesity; Af: above fat median; Bf: below fat median; BPA: bisphenol A; BPF: bisphenol F, BIS: total bisphenols.

To determine whether actual body fat distribution better captured this environmental burden, we next examined bisphenol exposure stratified by adiposity phenotypes (Figure 4). Strikingly, cumulative bisphenol exposure closely mirrored the SCFA trends seen previously. Normal-weight individuals with high adiposity (NW_Af) exhibited significantly higher concentrations of BPA (*p* = 0.011) and BIS (*p* = 0.031) compared to their lean counterparts (NW_Bf). While BPF levels did not differ significantly between the NW subgroups, a significant increase was observed between the OW and O groups (*p* = 0.048). Although these specific associations did not retain statistical significance after Benjamini-Hochberg (BH) adjustment due to the restricted sample size, the distinct dose-response-like pattern suggests a biologically relevant accumulation of bisphenols driven by body fat percentage, regardless of standard BMI classification.

### 3.5. Profiles of Gut Microbiota and SCFA Levels According to Bisphenol Levels

Having established the relationship between adiposity and bisphenol accumulation, we next assessed the impact of this environmental burden on the microbiota. Stratifying the cohort by median nail bisphenol concentrations (below (B) vs. above (A)), higher exposure was linked to distinct microbiota structural shifts, including increased microbial richness (Observed and Shannon indices, *p* < 0.05) and altered community composition (PERMANOVA, R^2^ = 0.04, *p* = 0.009; Supplementary Figure S5A,B). This above-median phenotype was characterized by widespread taxonomic enrichments, led by *Prevotella copri, Catenibacterium*, and *Parabacteroides goldsteinii* at the ASV level. Genus-level analyses confirmed these trends, showing marked enrichments in *Catenibacterium, Enterococcus*, and *Lactococcus*, alongside Desulfobacterota as the sole enriched phylum. Conversely, only the genus *Clostridium* CAG-352 was significantly depleted in the more exposed group (Supplementary Figure S5C–E).

**Figure 5:**
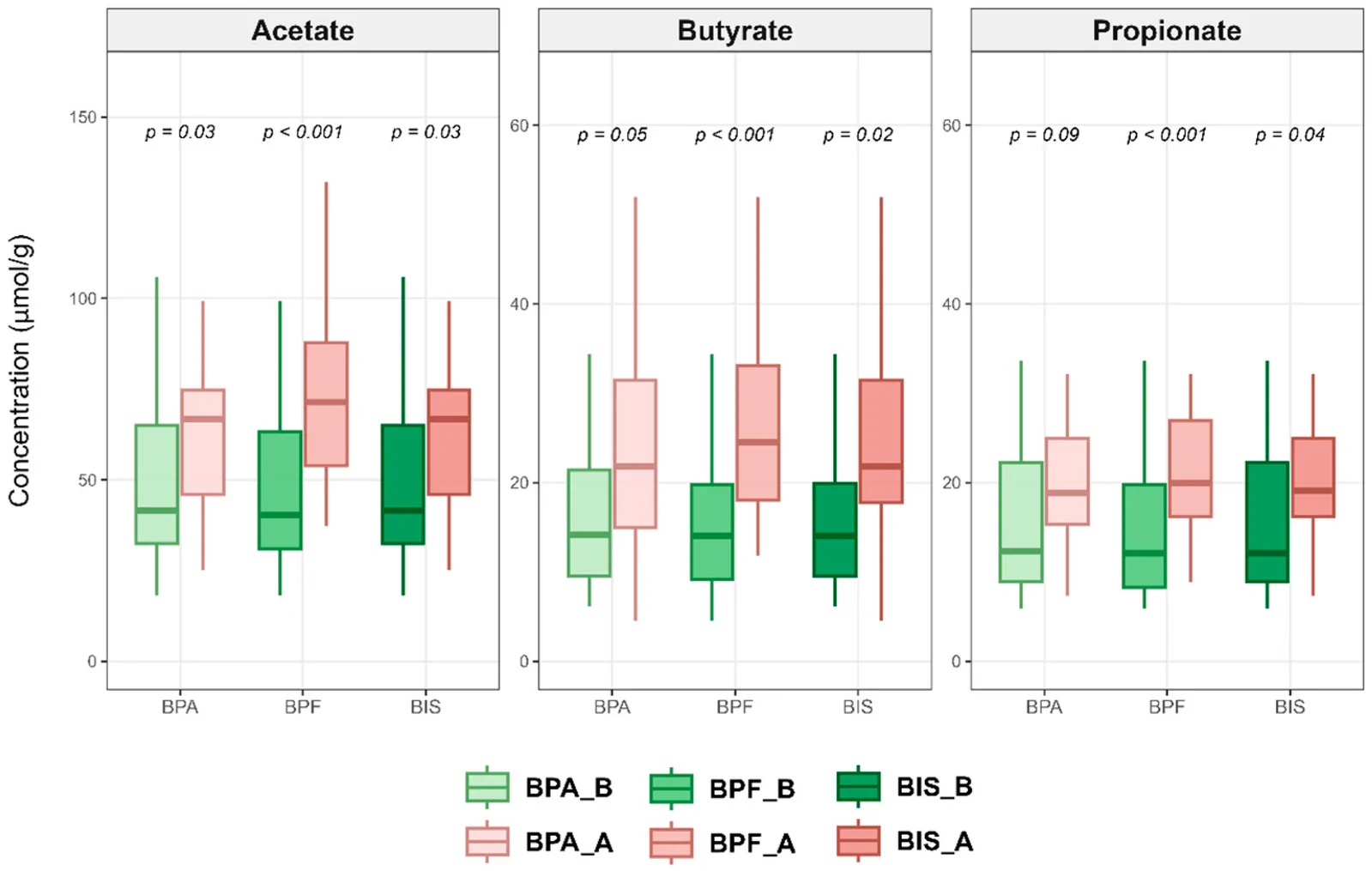
Fecal SCFA concentrations stratified by individual bisphenol exposure (bisphenol A, BPA; bisphenol F, BPF; total bisphenols, BIS) and further subdivided by median nail concentrations (Below median, _B; Above median, _A). Boxes represent interquartile ranges (IQR) with medians. Group differences were evaluated using Wilcoxon rank-sum tests with Benjamini–Hochberg FDR correction (adjusted *p*-values shown).

These microbiota perturbations in the more highly exposed individuals were accompanied by amplified targeted metabolite outputs. Fecal SCFA profiles (acetate, butyrate, and propionate) exhibited statistically significant increases in all above-median exposure groups (Figure 5).

### 3.6. Data Integration of SCFAs, Gut Microbiota Taxa, Bisphenols, and Obesity Indices

To better understand the complex interplay between the gut microbiota, bisphenol exposure, and host metabolic status, we conducted an integrative analysis focusing exclusively on core ASVs detected in at least 25% of the samples.

**Figure 6:**
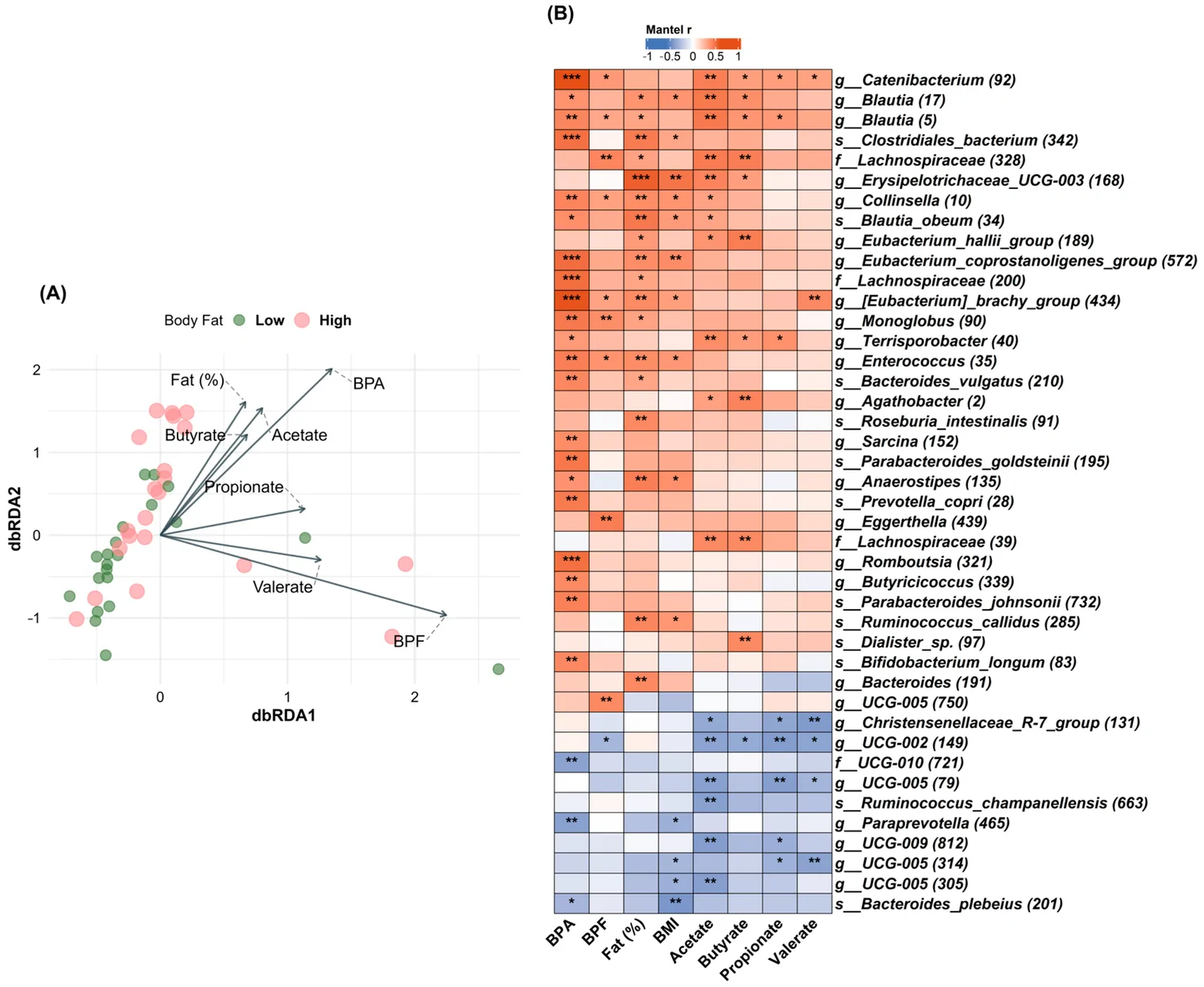
Integrative analysis of gut microbiota taxa, bisphenols, SCFAs and obesity indices. (**A**) Distance-based Redundancy Analysis (dbRDA) of microbiota composition based on Bray-Curtis dissimilarities. Samples (points) are colored and sized by Body Fat category. Vector arrows represent the direction and magnitude of the relationship between microbial community structure and the explanatory variables. (**B**) Heatmap displaying multivariate associations. Global community-level relationships were validated through Mantel tests using Spearman’s rank correlations (999 permutations). Only robust associations (r > 0.30; *p* < 0.01) are represented to facilitate data interpretation and clarity. All *p*-values are Benjamini-Hochberg FDR corrected (* *p* ≤ 0.05, ** *p* ≤ 0.01, *** *p* ≤ 0.001).

The multivariate relationship between environmental exposure, host metabolic status, and microbial community structure was first examined using distance-based Redundancy Analysis (dbRDA) (Figure 6A). The global model significantly explained the variance in microbial community structure (*p* = 0.03; adjusted R^2^ = 4.3%). While the overall variance explained is relatively low, as is typical for complex human microbiome ecosystems, the significant *p*-value confirms a consistent compositional pattern driven by host metabolic status and environmental exposure. Permutation tests for marginal effects identified nail BPF concentrations as a significant driver of community heterogeneity (*p* = 0.027), while BPA showed a borderline significant association (*p* = 0.058). Other variables, including body fat percentage and individual SCFAs, did not show significant independent contributions to the global model in the sequential ANOVA (all *p* > 0.05). Regarding the spatial orientation of the explanatory variables in the ordination plot, a notable co-directional alignment was observed among nail BPA concentrations, body fat percentage, and the SCFAs acetate and butyrate. These vectors are predominantly loaded onto the second axis (dbRDA2). In contrast, the first axis (dbRDA1) was characterized by the dominant influence of nail BPF, which exhibited the highest positive loading and shared this directional trajectory with propionate and valerate.

To further untangle these associations at the specific taxa level, we used Mantel tests and a multidimensional correlation heatmap (Figure 6B). This allowed us to identify specific microbial signatures driven by bisphenol exposure and adiposity. Several taxa were identified as part of an “exposure-adiposity” cluster. Notably, *Catenibacterium* (92) showed the strongest positive correlations with bisphenols, particularly BIS (r = 0.70) and BPA (r = 0.66), alongside consistent positive associations across all four primary SCFAs (acetate, butyrate, propionate, and valerate). The [*Eubacterium*] brachy group (434) and *Enterococcus* (35) exhibited robust positive links across the data spectrum, with the former strongly correlated to BIS (r = 0.60) and body fat percentage (r = 0.40). Taxa from the *Blautia* genus (ASVs 17, 5, and 34) and *Romboutsia* (321) also followed this trend, showing a synchronized increase with both bisphenol exposure and host adiposity markers. Conversely, an inverse cluster emerged, taxa members of the UCG-005 group (ASVs 314, 79, and 305) and UCG-009 (812) consistently showed negative correlations with bisphenols, body fat, and SCFAs. For instance, *Bacteroides plebeius* (201) was significantly depleted as BMI (r = −0.45) and BIS (r = −0.36) levels increased. Furthermore, the Christensenellaceae R-7 group (131) and UCG-002 (149) displayed strong inverse relationships with SCFA production, particularly with valerate (r = −0.43) and propionate (r = −0.42). 

### 3.7. Genomic Profiling of Microbial Dietary Fiber/Carbohydrate-Degradation Potential

To better understand the possible mechanisms contributing to the observed integrative associations between specific microbial taxa, high SCFA levels, bisphenol exposure, and host adiposity, we conducted a predictive functional genomic analysis targeting fiber and carbohydrate-degrading enzymes. Key ASVs, identified through the multidimensional correlation analysis, were screened for relevant Carbohydrate-Active enZymes (CAZymes) referencing the CAZy database. The genomic copy numbers of these enzymes were grouped into six major dietary polysaccharide substrates, representing key fermentable carbohydrate sources for microbial energy extraction and SCFA production, based on their specific catalytic affinities (Supplementary Table S5).

**Figure 7:**
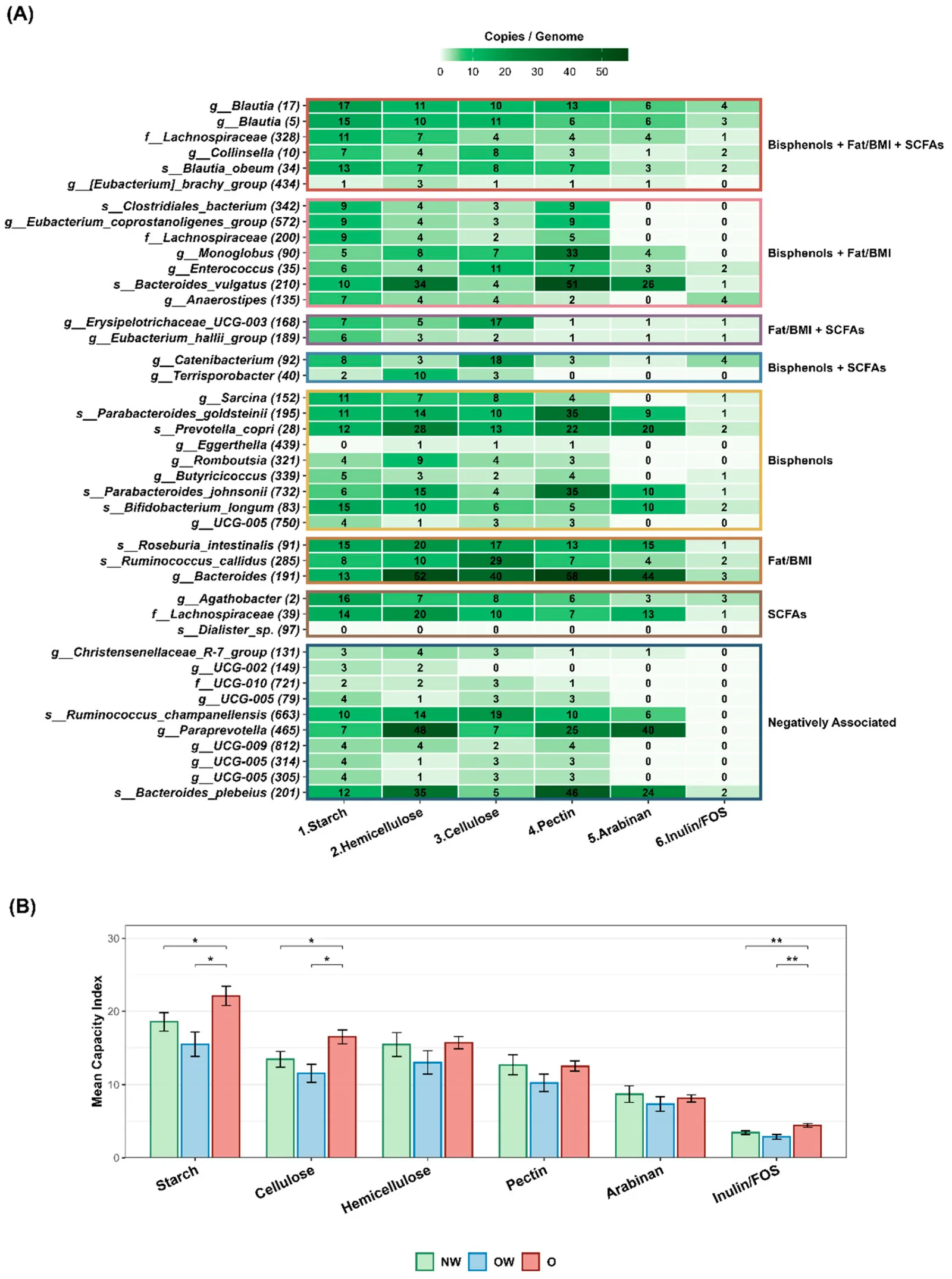
Dietary carbohydrate-degradation potential of ASVs associated with fecal SCFAs, adiposity, and bisphenol exposure. (**A**) Heatmap showing CAZy gene copies per reference genome across six major dietary carbohydrate substrates. Taxa are grouped according to their integrative associations with bisphenols, fat/BMI, and fecal SCFAs. (**B**) Mean abundance-weighted carbohydrate-degradation potential index of the selected ASVs across Normal-Weight (NW), Overweight (OW), and Obesity (O) groups. Error bars indicate standard error of the mean. Asterisks indicate significant pairwise differences (* *p* < 0.05, ** *p* < 0.01; Dunn’s test with Benjamini–Hochberg FDR correction). FOS: fructooligosaccharides.

Structuring these taxa based on their clinical and environmental association profiles revealed a functional divergence (Figure 7A). Taxa positively associated with adiposity markers, bisphenol exposure, or a combination of both, generally harbored elevated and redundant genomic copy numbers for carbohydrate degradation. For instance, *Blautia* (17) exhibited an extensive saccharolytic repertoire for complex carbohydrate and high number of inulinases, and *Bacteroides vulgatus* (210) displayed specialized redundancy for pectin degradation. Similarly, *Catenibacterium* (92), which clustered specifically with bisphenol exposure and elevated SCFAs, displayed specialized redundancy for cellulose and inulin. In contrast, the ‘negatively associated’ cluster, comprising taxa enriched in normal-weight and lower-exposure profiles, such as UCG-010, UCG-005, UCG-009, and the Christensenellaceae R-7 group, displayed minimal genomic redundancy. Notably, *Bacteroides plebeius* (201) and *Paraprevotella* (465) represented specialized, highly saccharolytic exceptions within this cluster, harboring massive genomic repertoires for hemicellulose and pectin degradation.

To assess the contribution of these differential taxa at the community level across BMI categories, an abundance-weighted carbohydrate-degradation potential index was calculated for each sample by weighting CAZy copy numbers by the relative abundance of the corresponding ASVs (Figure 7B). The predicted potential did not increase progressively with BMI but instead showed a non-linear, U-shaped pattern, with a decrease in the OW group followed by a significant increase in the O group (*p* < 0.05). Notably, while the absolute magnitude of the inulin/FOS degradation potential was the lowest among the substrates, it exhibited the most statistically variations across the BMI groups (*p* < 0.01). This increase coincided with higher abundances of taxa carrying redundant carbohydrate-degradation repertoires. A detailed breakdown of the specific contribution of each individual ASV to this community-level capacity across the BMI groups is provided in Supplementary Figure S6.

## 4. Discussion

Short-chain fatty acids (SCFAs) represent a major functional output of the gut microbiota and increasingly recognized as key mediators of host metabolic regulation. In agreement with previous pediatric studies [26], acetate, propionate, and butyrate were positively associated with BMI and body fat percentage in our cohort, whereas branched-chain fatty acids remained at the low concentrations typically reported in healthy children [27]. 

While SCFAs are traditionally recognized as beneficial signaling molecules with a documented range of health-promoting effects [9], our results suggest that their positive association with childhood obesity may reflect a complex, phenotype-dependent metabolic response. As proposed by Canfora et al. [28], metabolically compromised phenotypes, such as obese and prediabetic individuals, may exhibit a disruption in SCFA signaling pathways. Critically, the presence of environmental xenobiotics like bisphenols might be associated with a dysbiosis linked to a functional shift in SCFA utilization, potentially reflecting a shift toward energy extraction over regulatory signaling. In line with this, our findings showed that fecal SCFA levels, predominantly acetate, followed by butyrate, were significantly associated with obesity, adiposity, and bisphenol exposure. Current scientific evidence suggests that beyond their role as energy substrates, SCFAs, predominantly acetate and propionate, function as potent signaling ligands in white adipose tissue via the GPR43 (FFAR2) receptor, which has been shown to up-regulate the expression of PPARγ, the master regulator of adipogenesis, to enhance lipid accumulation while suppressing triglyceride breakdown [29,30]. Furthermore, as reported by Perry et al. [31], increased acetate production can activate the parasympathetic nervous system, leading to heightened secretion of ghrelin and glucose-stimulated insulin secretion (GSIS), potentially contributing to a feedback loop associated with hyperphagia and fat deposition. Strikingly, bisphenols exert a parallel effect by directly triggering preadipocyte differentiation and lipid storage through the induction of PPARγ [32]. Consequently, the concurrent elevation of microbial SCFAs and bisphenols in high-adiposity children might point toward a synergistic contribution to fat accumulation.

From a host–microbiome perspective, the interpretation of elevated fecal SCFAs requires careful considerations. Both systemic and fecal SCFA levels may underestimate or misrepresent actual microbial production, as measured concentrations can be strongly influenced by gut permeability, colonic absorption and splanchnic metabolism [33]. Thus, the higher fecal SCFA concentrations observed in children with obesity may indicate impaired proximal absorption rather than solely increased microbial biosynthesis, leading to prolonged luminal persistence of these metabolites. Furthermore, optimal SCFA output relies on coordinated consortia of primary degraders and cross-feeders [34]. In our cohort, taxonomic analyses revealed a profound disruption of this orchestrated balance. Specifically, children with obesity and high bisphenol exposure exhibited a targeted depletion of specialized homeostatic degraders, such as the Christensenellaceae R-7 group, Oscillospiraceae clades, and *Bacteroides plebeius*, which are classically associated with metabolic health [35,36,37]. Simultaneously, we observed the expansion of a highly fermentative consortium across multiple phyla. This exposure- and adiposity-associated taxa group includes ASVs from *Blautia, Bacteroides vulgatus, Prevotella copri*, alongside *Catenibacterium*, which emerged as a key taxon strongly associated with bisphenol concentrations and elevated SCFAs. Specially, *Catenibacterium* and *Blautia* (17) harbored high genomic copy numbers for inulinases and correlated positively across all measured adiposity, exposure, and metabolic variables. This enhanced genomic capacity for inulin fiber fermentation is consistent with greater volatile fatty acid production, a pattern previously associated with increased obesity risk [38]. Supporting this ecological interpretation, our predictive functional analysis revealed a non-linear, U-shaped pattern in the polysaccharide-degradation potential of the key differential ASVs across BMI categories. This potential decreased in overweight children, however increased in obesity, particularly for starch, cellulose, hemicellulose and inulin degradation. Taxa positively associated with host metabolic risk factors, bisphenol exposure, and/or fecal SCFAs generally exhibited greater predicted genomic potential for dietary polysaccharide degradation than taxa associated with normal-weight and lower-bisphenol exposure profiles. Conversely, the latter group generally showed lower saccharolytic potential, with exceptions of specialized degraders such as *Bacteroides plebeius*, *Paraprevotella*, and *Ruminococcus champanellensis* [39,40]. The predictive functional pattern is consistent with the possibility that the obesity-associated microbial community harbor an enhanced capacity to access and degrade complex dietary carbohydrates, suggesting an association with greater microbial energy-harvesting potential and increased availability of fermentation-derived metabolites to the host [8,41]. 

It is important to acknowledge that, given the cross-sectional design of this study, reported associations do not allow causal relationships or mechanistic inferences to be established. Additionally, bisphenol analyses were restricted to a subset of 42 children for whom nail matrix biomonitoring data were available. While this subset demonstrated a high compositional concordance with the full cohort, the reduced sample size inherently limits statistical power. Regarding the nail matrix, although it provides a non-invasive and temporally integrated measure of chronic bisphenol exposure, sample collection difficulties in pediatric populations and potential contamination should be considered as inherent limitations of this biomonitoring approach. Furthermore, the functional analyses presented here rely on predictive CAZyme profiling inferred from 16S amplicon data using reference genome databases, rather than direct metagenomic measurements that will be performed in near future. Longitudinal and larger studies integrating dietary tracking, systemic sampling, and multi-omics approaches are essential to address these limitations and to further elucidate and support the true mechanisms and dynamics underlying the ‘chemical obesogen–microbiome’ interplay. 

## 5. Conclusions

Collectively, our taxonomic, metabolic, environmental, and predicted functional analyses and outcomes converge toward a common dysbiosis model. Childhood adiposity was characterized by elevated fecal SCFAs, expansion of fermentative bacterial taxa, depletion of health-associated clades, enrichment of predicted carbohydrate-active enzymes, and a parallel pattern of bisphenol exposure. Although causal relationships cannot be directly established from this study, the convergence of these independent data layers strongly supports the hypothesis that childhood obesity is associated with a specific altered gut microbial ecosystem displaying enhanced fermentative and energy-harvesting potential. These findings further suggest that chronic exposure to endocrine-disrupting chemicals such as bisphenols may contribute to this microbial dysbiosis, reinforcing the concept of dietary contaminant–microbiome–adiposity linkage.

## Figures and Tables

**Table 1:** General characteristics of the pediatric cohort included in the study. Median and Interquartile Range [IQR, 25th percentile–75th percentile].

	NW (*n* = 47)	OW (*n* = 16)	O (*n* = 37)	*p* Value
Female (%)	60.4	62.5	35.1	0.0461 ^1^
Age	8 [6, 9]	10 [7, 10]	8 [7, 10]	0.2372 ^2^
BMI	15.80 [14.67, 16.80]	19.95 [18.52, 21.35]	24.60 [22.30, 25.90]	<0.0012 ^2^
Fat (%)	20.45 [17.80, 22.63]	26.25 [25.03, 28.70]	33.50 [30.60, 38.30]	<0.0012 ^2^

^1^ Pearson’s Chi-squared test; ^2^ Kruskal-Wallis test.

## Data Availability

Data will be made available on request.

## Supplementary Materials

The following supporting information can be downloaded at: https://www.mdpi.com/article/10.3390/nu18213560/s1, Table S1: Mass/charge (*m*/*z*) of target ion (TI), confirmative ion 1–2 (CI1, CI2), retention time (RT), and deuterated internal standard for analysed SCFAs; Table S2: Median concentrations and interquartile ranges [IQR] of fecal SCFAs (μmol/g) in the study population; Table S3: General characteristics and concentration of bisphenol A (BPA), bisphenol F (BPF), and total bisphenols (BIS) in nails (ng/g) of the participant subset; Table S4: Median of fecal SCFAs (μmol/g) in the participant subset; Table S5: Dietary polysaccharide substrate categories and corresponding CAZy families used to estimate predicted degradation potential; Figure S1: Bubble-forest plot representing Spearman’s correlation between fecal SCFA concentrations and differentially abundant ASVs; Figure S2: Evaluation of gut microbiota representativeness between the full cohort and the bisphenol subcohort; Figure S3: Global overview of gut microbiota composition of the participant subset included in the nail bisphenol biomonitoring analysis; Figure S4. Concentrations of individual and total bisphenols in nails across BMI categories; Figure S5. Global overview of gut microbiota composition in relation to bisphenol exposure; Figure S6: ASV-specific contributions to community carbohydrate-degrading capacity across childhood BMI categories..

## Abbreviations

The following abbreviations are used in this manuscript:

ASV	Amplicon Sequence Variant
BPA	Bisphenol A
BPF	Bisphenol F
CAZyasvme	Carbohydrate-Active enZyme
CI	Confirmative ion
dbRDA	distance-based Redundancy Analysis
EDCs	Endocrine-disrupting chemicals
GC-MS	Gas Chromatography coupled to Mass Spectrometry
IQR	Interquartile Range
PCoA	Principal Coordinates Analysis
SCFA	Shot-Chain Fatty Acids
SD	Standard Deviation
TI	Target ion
