# Supplementary material for: Childhood Adiposity and Bisphenol Exposure Are Linked to Gut Dysbiosis, Altered Short-Chain Fatty Acid and Complex Carbohydrate-Degrading Profiles
Source: Nutrients. 2026 Oct 28;18(21):3560. doi: 10.3390/nu18213560 (PMC13764833; doi:10.3390/nu18213560)
Supplement: Supplementary file 1 [file nutrients-18-03560-s001.zip › nutrients-4631295-supplementary.pdf]

**Supplementary Table S1**. Mass/charge (m/z) of target ion (TI), confirmative ion 1–2 (CI1, CI2), retention time (RT), and deuterated internal standard for analysed SCFAs.

| SCFA | RT | m/z (TI) | m/z (CI1, CI2) |
|---|---|---|---|
| Acetate | 5.9 | 43, 45, 60 | 45, 60 |
| Propionate | 7 | 74, 45, 57 | 45, 57 |
| Isobutyrate | 7.44 | 43, 73, 88 | 73, 88 |
| Butyric Acid d7 | 8.27 | 63, 77, 46 | 77, 46 |
| Butyrate | 8.42 | 60, 41, 73 | 41, 73 |
| Isovalerate | 9.33 | 60, 43, 87 | 43, 87 |
| Valerate | 10.27 | 60, 41, 73 | 41, 73 |
| Methyl Valerate | 10.82 | 57, 43, 74 | 43, 74 |
| Hexanoic Acid | 11.09 | 60, 73, 87 | 73, 87 |

**Supplementary Table S2.** Median concentrations and interquartile ranges [IQR] of fecal SCFAs (μmol/g) in the study population.

| SCFA (μmol/g) | NW | OW | O |
|---|---|---|---|
| Acetate | 47.11 [34.00, 66.35] | 46.22 [35.51, 59.76] | 74.65 [56.10, 102.40] |
| Butyrate | 17.63 [11.16, 21.29] | 15.08 [9.86, 21.03] | 25.95 [16.94, 38.46] |
| Propionate | 14.97 [10.55, 20.47] | 12.89 [10.74, 20.41] | 21.91 [16.57, 26.99] |
| Valerate | 3.70 [2.55, 4.54] | 3.19 [2.48, 4.08] | 4.79 [3.42, 7.84] |
| Isobutyrate | 3.07 [2.07, 3.76] | 2.94 [2.32, 3.35] | 3.57 [2.49, 5.05] |
| Isovalerate | 2.44 [1.79, 2.94] | 2.26 [1.77, 2.93] | 3.40 [1.84, 4.72] |

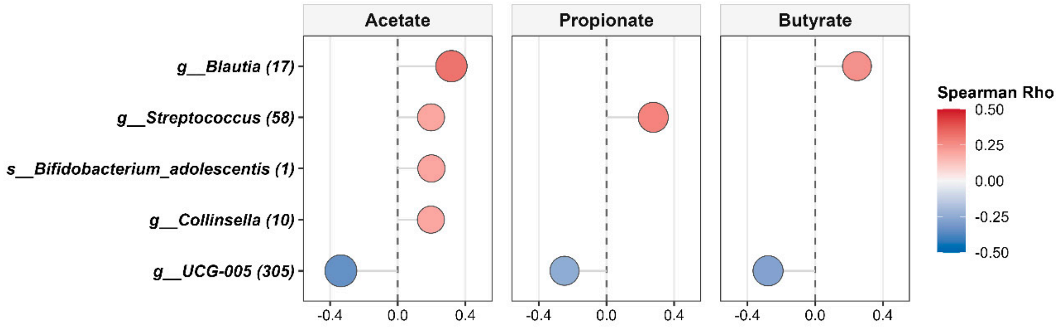


**Supplementary Figure S1.** Bubble-forest plot representing Spearman's correlation between fecal SCFA concentrations and differentially abundant ASVs. Bubble color indicates the direction of the association, with red and blue representing positive and negative correlations, respectively. The size of the bubbles reflects the magnitude of the Spearman's rho coefficient. All displayed correlations are statistically significant after Benjamini-Hochberg adjustment ($p < 0.05$).

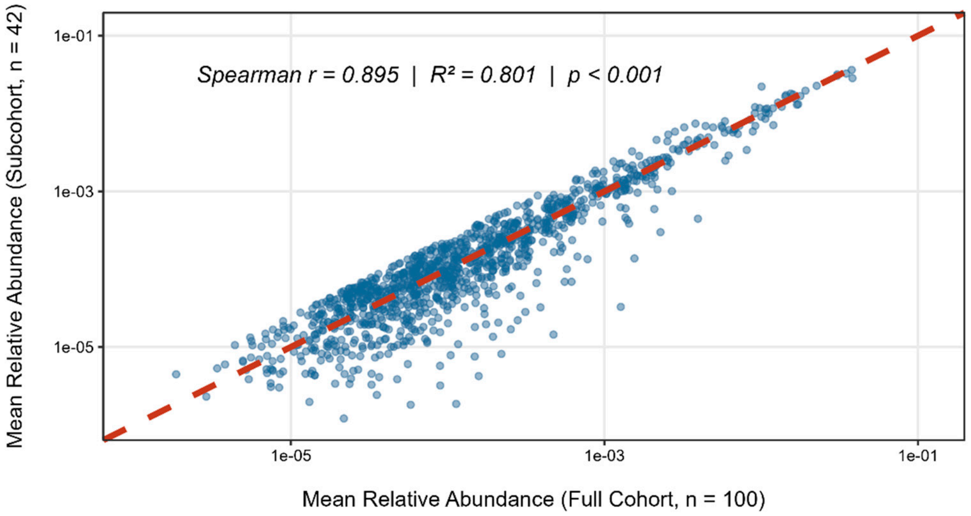


**Supplementary Figure S2.** Evaluation of gut microbiota representativeness between the full cohort and the bisphenol subcohort. Scatter plot illustrating the correlation of mean relative abundances of ASVs between the full study population (n=100, x-axis) and the nail bisphenol subcohort (n=42, y-axis). Each blue dot represents an individual ASV plotted on a logarithmic scale. The red dashed line denotes perfect 1:1 identity (y = x). Statistical concordance was evaluated using Spearman's rank correlation coefficient ($r$ = 0.895, $R^2$ = 0.801, $p < 0.001$).

**Supplementary Table S3.** General characteristics and concentration of bisphenol A (BPA), bisphenol F (BPF), and total bisphenols (BIS) in nails (ng/g) of the participant subset. Median values and interquartile ranges [IQR].

| | NW (n = 25) | OW (n = 8) | O (n = 9) | *p* value |
|---|---|---|---|---|
| **Female (%)** | 52 | 87.5 | 22.2 | 0.03[1] |
| Age | 8.00<br>[6.00, 9.00] | 10.00<br>[7.75, 10.25] | 8.00<br>[6.00, 9.00] | 0.30[2] |
| BMI $kg/m^2$ | 16.01<br>[15.00, 17.00] | 20.10<br>[19.08, 22.42] | 23.00<br>[21.20, 25.40] | <0.001[2] |
| Fat (%) | 20.20<br>[18.00, 22.60] | 27.75<br>[26.47, 30.65] | 33.20<br>[32.40, 33.60] | <0.001[2] |
| BPA | 43.87<br>[25.48, 622.30] | 75.75<br>[14.91, 376.45] | 547.60<br>[39.42, 1073.30] | 0.32 |
| BPF | 10.55<br>[6.95, 34.45] | 8.30<br>[5.06, 21.06] | 26.80<br>[18.56, 54.10] | 0.09 |
| BIS | 57.54<br>[37.81, 688.60] | 83.04<br>[28.18, 538.15] | 568.60<br>[82.10, 1178.10] | 0.26 |

IQR: interquartile range (25th percentile–75th percentile). [1]Pearson's Chi-squared test; [2]Kruskal-Wallis test.

**Supplementary Table S4.** Median of fecal SCFAs (µmol/g) in the participant subset. NW_B: normal-weight below fat median; NW_A: normal-weight above fat median; OW_Bf: overweight below fat median; OW_Af: overweight above fat median; O_Af: obesity above fat median

| BMI | n | Acetate | Butyrate | Propionate |
|---|---|---|---|---|
| NW_Bf | 44 | 47.1 | 17.6 | 14.7 |
| NW_Af | 4 | 57.8 | 26.1 | 16.3 |
| OW_Bf | 5 | 43.5 | 14.5 | 12.2 |
| OW_Af | 11 | 48.9 | 12.0 | 13.4 |
| O_Af | 37 | 74.6 | 26.0 | 21.9 |

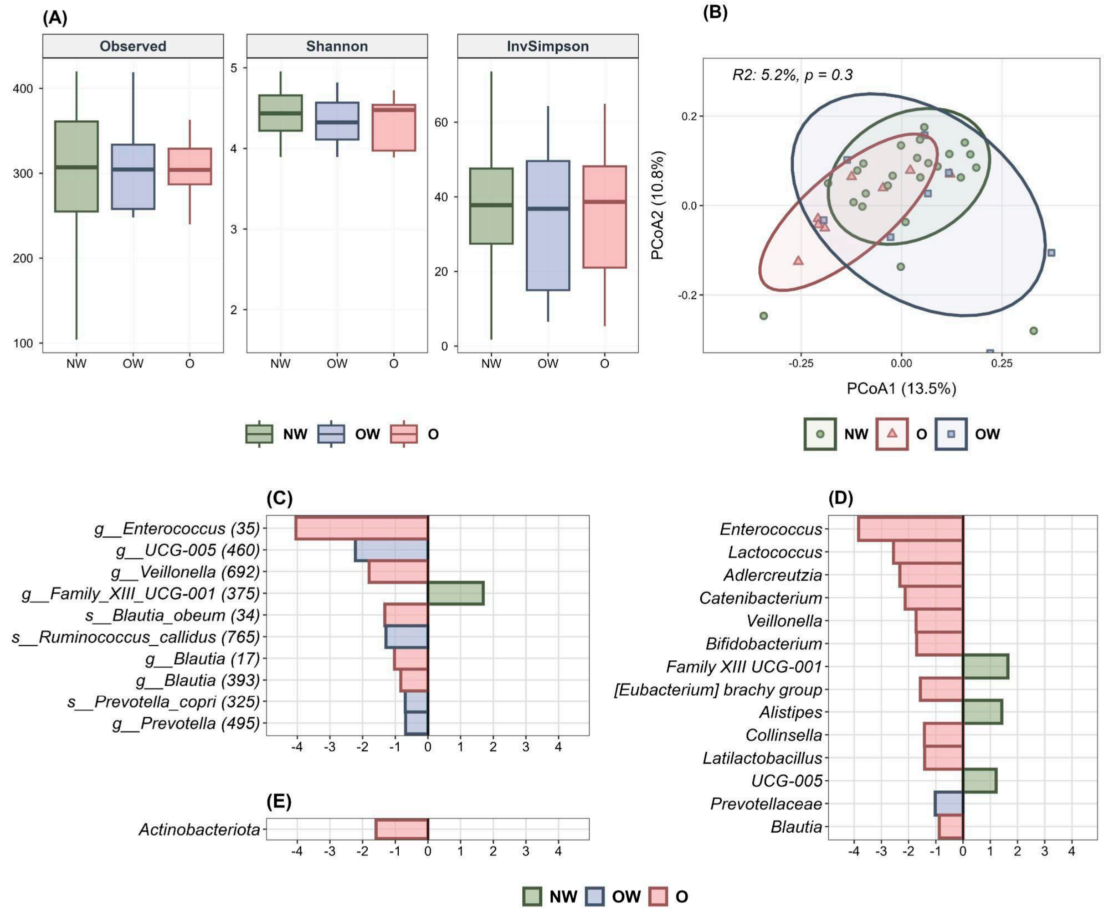


**Supplementary Figure S3.** Global overview of gut microbiota composition of the participant subset included in the nail bisphenol biomonitoring analysis. (A) Alpha diversity indices including Observed, Shannon, and Inverse Simpson. (B) Principal Coordinate Analysis (PCoA) based on Bray-Curtis dissimilarity showing the microbial community composition (beta diversity) of the study cohort. Differentially abundant ASVs (C), genera (D), and phylum (E) in association with BMI. Bar plots depict MaAsLin2 analysis results adjusted for sex. Significant results are shown (q < 0.1). Bar lengths correspond to the model coefficient, which relates to the strength of the association. NW: normal weight, OW: overweight; O: obesity.

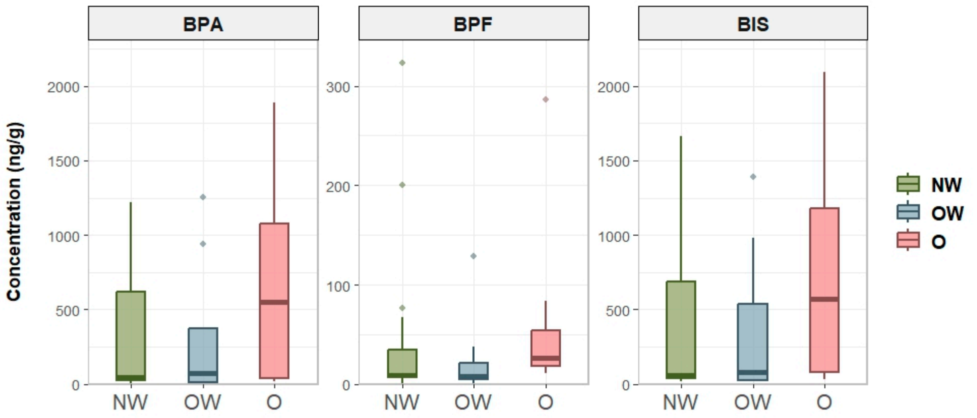


**Supplementary Figure S4.** Concentrations of individual and total bisphenols in nails across BMI categories. BPA: bisphenol A; BPF: bisphenol F; BIS: total bisphenols; NW: normal weight; OW: overweight; O: obesity.

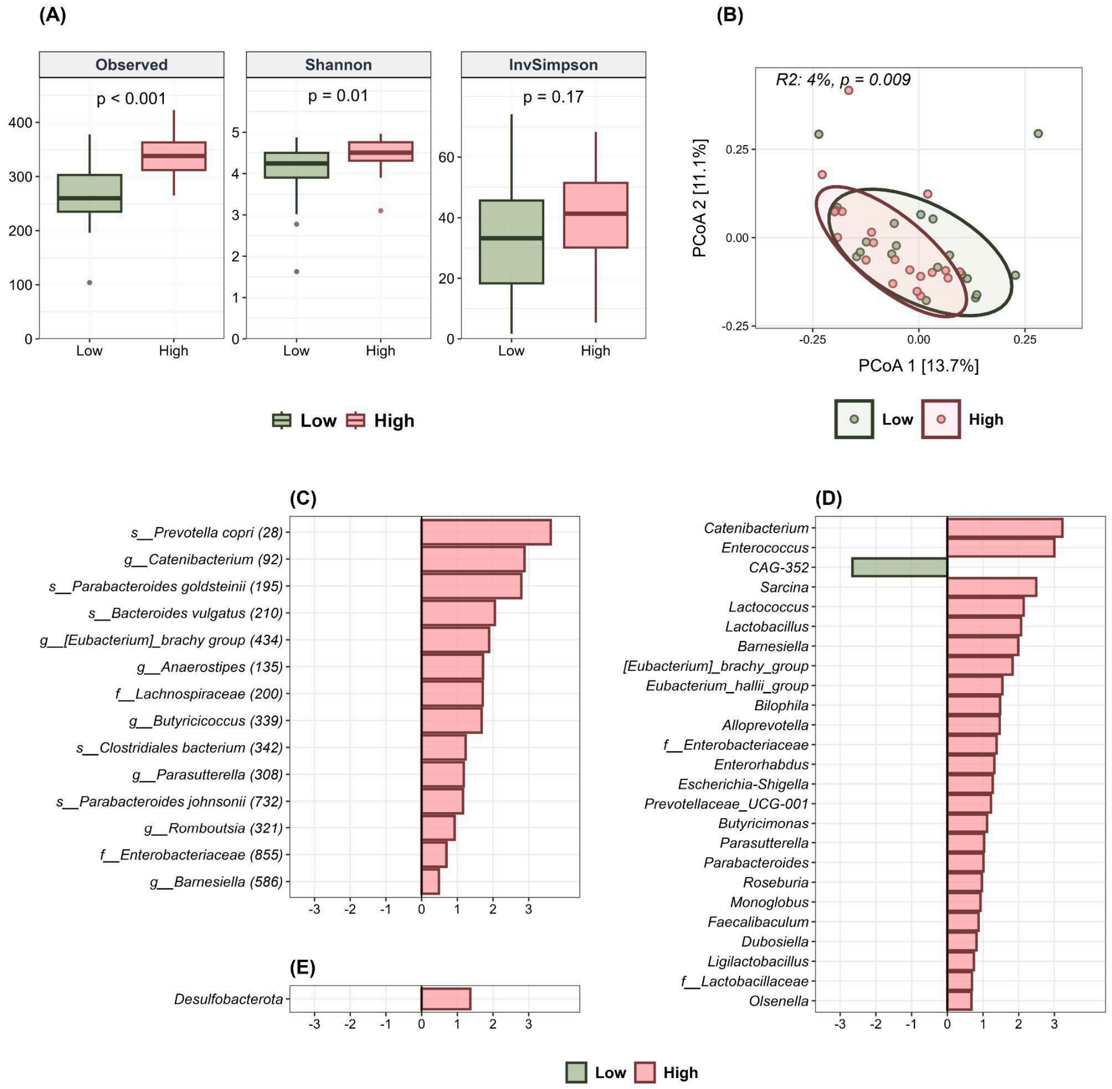


**Supplementary Figure S5.** Global overview of gut microbiota composition in relation to bisphenol exposure. (A) Alpha diversity indices including Observed, Shannon, and Inverse Simpson. (B) Principal Coordinate Analysis (PCoA) based on Bray-Curtis dissimilarity showing the microbial community composition (beta diversity) of the study cohort. Differentially abundant ASVs (C), genera (D), and phylum (E) in association with bisphenol levels. Bar plots depict MaAsLin2 analysis results adjusted for sex.

Significant results are shown (q < 0.1). Bar lengths correspond to the model coefficient, which relates to the strength of the association.

**Supplementary Table S5.** Dietary polysaccharide substrate categories and corresponding CAZy families used to estimate predicted degradation potential.

| Target substrate | CAZy families included | Representative enzyme activities |
|---|---|---|
| Starch | GH13, GH14, GH15, GH31, GH57, GH77, GH97 | α-amylases, β-amylases, glucoamylases, α-glucosidases, glucanotransferases and starch-debranching enzymes |
| Inulin and fructooligosaccharides (FOS) | GH32, GH68, GH91 | Endo-inulinase, exo-inulinase, fructan β-fructosidase, β-fructofuranosidase, inulosucrase, levansucrase, fructan 1-exohydrolase-like activity |
| Cellulose | GH1, GH3, GH5, GH8, GH9, GH44, GH45, GH48, GH74 | β-glucosidases, endoglucanases and cellobiohydrolases/exoglucanases |
| Hemicellulose | GH3, GH10, GH11, GH26, GH30, GH43, GH51, GH53 | Xylanases, xylosidases, mannanases, mannosidases and galactanases |
| Arabinan | GH43, GH51, GH62, GH93 | α-L-arabinofuranosidases and endo-/exo-arabinanases |
| Pectin | GH2, GH28, GH78, GH88, GH105, PL1, PL2, PL9, PL11, CE8, CE12 | Rhamnogalacturonan-degrading enzymes, polygalacturonases, pectate/pectin lyases and esterases |

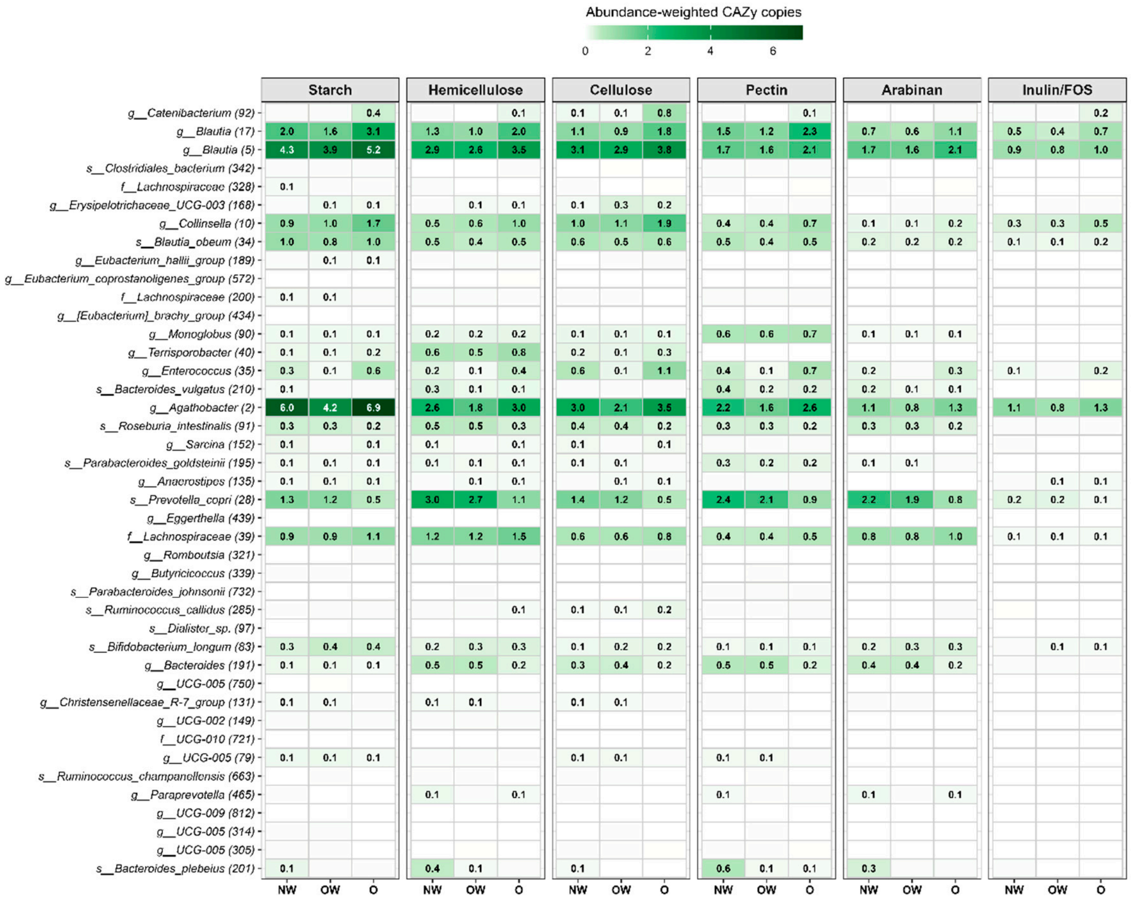

**Supplementary Figure S6.** ASV-specific contributions to community carbohydrate-degrading capacity across childhood BMI categories. Heatmap displaying the mean abundance-weighted CAZy gene copies of the targeted differential ASVs across six major dietary carbohydrate substrates. The contribution value for each ASV was calculated by weighting its sample-level relative abundance by its genomic CAZy copy number for the respective enzymatic category, averaged across Normal-Weight (NW), Overweight (OW), and Obesity (O) groups. For visual clarity, only contribution values ≥ 0.1 are displayed.
